# Goreisan Inhibits Upregulation of Aquaporin 4 and Formation of Cerebral Edema in the Rat Model of Juvenile Hypoxic-Ischemic Encephalopathy

**DOI:** 10.1155/2017/3209219

**Published:** 2017-10-18

**Authors:** Yoshiaki Yano, Hajime Yano, Hisaaki Takahashi, Kouhei Yoshimoto, Shinji Tsuda, Kenta Fujiyama, Yusuke Izumo-Shimizu, Ryota Motoie, Masanori Ito, Junya Tanaka, Eiichi Ishii, Mitsumasa Fukuda

**Affiliations:** ^1^Department of Pediatrics, National Hospital Organization Ehime Medical Center, Yokogawara, Toon, Ehime, Japan; ^2^Department of Pediatrics, Ehime University Graduate School of Medicine, Shitsukawa, Toon, Ehime, Japan; ^3^Department of Molecular and Cellular Physiology, Ehime University Graduate School of Medicine, Shitsukawa, Toon, Ehime, Japan; ^4^Division of Pathophysiology, Faculty of Pharmaceutical Sciences, Hokuriku University, Ho-3, Kanagawamachi, Kanazawa, Ishikawa, Japan

## Abstract

Secondary cerebral edema regulation is of prognostic significance in hypoxic-ischemic encephalopathy (HIE), and aquaporin 4 (AQP4) plays an important role in the pathogenesis of cerebral edema. The traditional Japanese herbal medicine Goreisan relieves brain edema in adults; however, its effect and pharmacological mechanism in children are unknown. We investigated the effects of Goreisan on HIE-associated brain edema and AQP4 expression in a juvenile rat model, established by combined occlusion of middle cerebral and common carotid arteries. Magnetic resonance imaging showed that the lesion areas were significantly smaller in the Goreisan- (2 g/kg) treated group than in the nontreated (saline) group at 24 and 48 h postoperatively. AQP4 mRNA levels in the lesion and nonlesion sides were significantly suppressed in the Goreisan group compared with the nontreated group 36 h postoperatively. Western blotting revealed that levels of AQP4 protein were significantly decreased in the Goreisan group compared with the nontreated group in the lesion side 72 h postoperatively, but not at 12 or 36 h. After 14 days, the Goreisan group had a significantly better survival rate. These findings suggest that Goreisan suppresses brain edema in HIE and improves survival in juvenile rats, possibly via regulation of AQP4 expression and function.

## 1. Background and Introduction

Hypoxic-ischemic encephalopathy (HIE) in children occurs in many settings and often has devastating neurological sequelae. Hypoxic-ischemic events before or around the time of birth occur in approximately 1 to 6 per 1000 live full-term births and are associated with a high risk of death or lifelong disability. In older children, acute encephalopathy/encephalitis, drowning, asphyxiation, trauma, and cardiac arrest are common causes of HIE, and the outcomes of such insults ultimately lead to difficult-to-treat brain damage. Moreover, cardiac arrest occurs at a frequency of 8 to 20 per 100,000 children in the United States [1].

Management of children with HIE is limited to supportive intensive care without specific therapy for the brain. Clinical evidence indicates that mildly induced hypothermia significantly improves survival and ameliorates disabilities, such as cerebral palsy and impaired cognition, of full-term infants with moderate-to-severe HIE [2–4], but it generally shows a partial effect. Stem cell therapy is a candidate for treating HIE. For example, intravenous transplantation of human umbilical cord-blood mesenchymal stem cells may be efficacious for humans because these cells improve the recovery of neurological function in rats with HIE [5, 6]. A clinical trial using umbilical cord-blood cells administered to neonates with HIE indicates the safety and feasibility of this stem cell therapy [7]. However, the use of stem cells for hypoxic-ischemic brain injury is still in the experimental phase. Therefore, effective new therapies are urgently required to treat patients with HIE.

Severe HIE is accompanied by cerebral edema, and its effects are compounded by increased intracranial pressure. Abnormal accumulation of water in the brain (cerebral edema) is a major clinical problem. Brain edema is associated with many neurological disorders, such as brain tumors and abscesses, meningitis, and head injury, and with extracranial pathologies that secondarily affect the brain, such as liver failure and sepsis. During the hypoxic-ischemic event, which can be the primary phase of cell injury, high-energy metabolites are rapidly depleted, leading to cytotoxic cell edema [8]. After the return of cerebral circulation and oxygenation, the initial hypoxia-induced cerebral edema and accumulation of excitatory amino acids typically resolve over approximately 30 to 60 min, with at least partial recovery of cerebral oxidative metabolism during the latent phase [9]. However, cerebral oxidative metabolism may then deteriorate many hours later and extend over many days. This secondary deterioration is often marked by the onset of secondary vasogenic edema-associated disruption of the blood-brain barrier (BBB), accumulation of excitotoxins, failure of cerebral mitochondrial activity, and cell death [8, 10, 11]. Detailed knowledge of the mechanisms of HIE and the regulation of cerebral edema will be required to develop more effective neuroprotective interventions.

Goreisan is a traditional Japanese herbal medicine that effectively relieves cerebral edema of adults. Goreisan is a mixture of five herbs, including Alismatis Rhizome,* Atractylodes lancea* Rhizome, Polyporus Sclerotium, Poria Sclerotium, and cinnamon bark (Table 1). A retrospective study indicates that Goreisan reduces the requirement for a second operation after burr-hole surgery for chronic subdural hematoma via the regulation of cerebral edema [12]. However, there is no clinical evidence for the effectiveness of Goreisan for treating brain edema-associated HIE. During the formation of cerebral edema, the water channel aquaporin 4 (AQP4) facilitates astrocyte swelling, and AQP4 mediates the reabsorption of extracellular edema fluid [13]. Therefore, the regulation of AQP4 expression may be effective for ameliorating the effects of severe HIE.

The AQP4 protein level is dependent on the time course after HIE. Specifically, after rats are subjected to ischemic injury, AQP4 levels increase and peak 6–12 h after injury, which is called the “acute” phase, then decrease to minimum during the “subacute” phase at approximately 24 h, and finally increase again to peak at approximately 72 h, which is called the “chronic” phase [14]. The first and second peaks may contribute to cytotoxic cellular edema and vasogenic edema, respectively [15]. In contrast, after injury, AQP4 mRNA is expressed at a higher level than normal at 24 h [16].

Most studies on HIE have been performed in neonatal or adult rats, but not in juvenile rats. Importantly, brain blood flow and the pathogenesis of ischemic brain injury are profoundly affected by the maturity of the brain at the time of the insult [17–19]. Therefore, an age-appropriate animal model associated with the maturity of the central nervous system should be established to clarify the pathogenesis of HIE. Here we used juvenile rats to study the effects of Goreisan on juvenile HIE and on the regulation of AQP4 expression associated with HIE.

## 2. Materials and Methods

### 2.1. Animals

Male Wistar rats (Charles River Laboratories Japan, Yokohama, Japan) were kept in a quiet, temperature-controlled room under a 12 h light-dark cycle, with freely available food and water. This study was carried out in strict accordance with the recommendations in the Guide for the Care and Use of Laboratory Animals of the Ministry of Education of Japan. The protocol was approved by the Committee on the Ethics of Animal Experiments of Ehime University (Permission Number: 05TE23-2). All surgeries were performed under isoflurane or ethyl ether anesthesia, and all efforts were made to minimize suffering.

### 2.2. Establishment of Hypoxic-Ischemic Encephalopathy Model and Drug Administration

The juvenile HIE rat model was established by middle cerebral artery occlusion (MCAO) together with common carotid artery occlusion (CCAO). We used 21-22-day-old rats (P21-22) (weighing 45–55 g). Rat brain development at P 21-22 corresponds to that of the juvenile human brain; therefore, the 21-22-day-old rat is an appropriately aged model for studying juvenile convulsive disorders [20]. These rats were anesthetized using isoflurane (2%) and a midline incision was made in the neck. The right CCA and external carotid artery (ECA) were isolated. Using bipolar forceps, the ECA was dissected and reversed. Following ligation of the CCA with 5-0 surgical silk, a microvascular clip was placed on the ECA-internal carotid artery (ICA) junction. A small incision was made in the distal end of the ECA stump and a 5-0 thread with a coating at the tip was inserted into the ICA from the ECA. The thread was advanced to the microvascular clip, which was then removed, and the thread was advanced 2 cm into the ICA to transiently occlude the MCA. The thread was removed after 60 min and the ECA was immediately permanently ligated with 5-0 surgical silk. Finally, the CCA was dissected using bipolar forceps. During the procedure, we maintained body temperature at a normal level using a heating pad.

**Figure 1:**
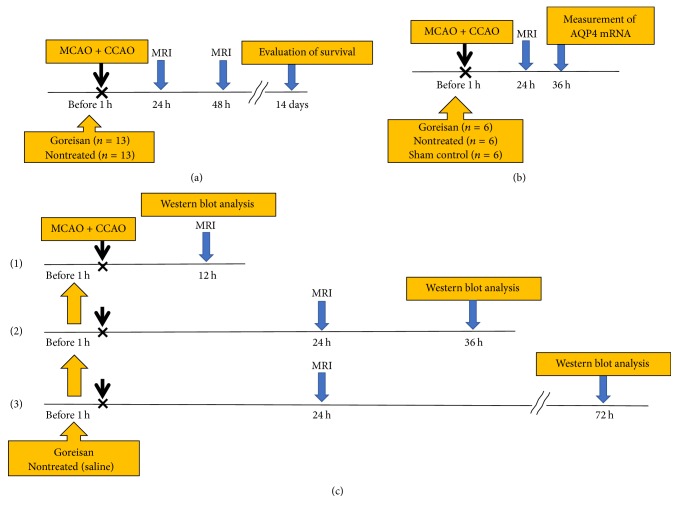
*Experimental procedure and design*. (a) Rats were divided into Goreisan and nontreated groups. Rats in the Goreisan group received Goreisan (2.0 g/kg) dissolved in saline (total volume 400 *μ*l) administered orally 1 h before surgery. Rats in the nontreated group received saline. MRI [T2-weighted (T2w) and diffusion-weighted (DW)] was performed at 24 and 48 h, and the survival rate was evaluated 14 days after surgery. The infarcted area at the maximum incised surface was observed using MRI (DW and T2w). We calculated the comparative amounts of the infarcted areas using the formula of Swanson et al. [22]. (b) An MRI was performed at 24 h, and the brain was removed at 36 h to evaluate AQP4 expression using immunoblotting and quantitative reverse-transcription PCR. (c-(1)) We examined AQP4 protein levels using western blot analysis at 12 h after MCAO + CCAO for the acute phase. (c-(2)) MRI was performed at 24 h and the brain was dissected at 36 h for the subacute phase. (c-(3)) MRI was performed at 24 h and the brain was dissected at 72 h for the chronic phase.

On P21-22, rats were divided into the Goreisan-treated ischemic group (Goreisan group, *n* = 13) or nontreated ischemic group (nontreated group, *n* = 13; Figure 1(a)). Goreisan (2.0 g/kg; Tsumura, Tokyo, Japan) was dissolved in saline up to a total volume of 400 *μ*l and administered orally to the rats in the Goreisan group 1 h before the surgery. Rats in the control group received an equal volume of saline. The appropriate Goreisan dose for humans is 0.2 g/kg. We used a dose that was tenfold used in humans (2 g/kg), with reference to a human equivalent dose [21]. All experimental rats could move and displayed hemiparesis 12 hours after surgery; no differences were observed between Goreisan and control groups. We regulated body temperature (35–37°C) after the operation and Goreisan did not affect body temperature.

### 2.3. Magnetic Resonance Imaging

Magnetic resonance imaging (MRI) was performed 24 and 48 h after surgery. Rats were lightly anesthetized using isoflurane (2.0%) and imaged using an MR mini SA 1506 1.5 T MRI (DS Pharma Biomedical Co., Ltd., Osaka, Japan). T2-weighted (T2w) imaging and diffusion-weighted imaging (DWI) sequences were acquired (20 coronal slices, 1 mm thick, 1 mm interleaves). The infarcted area at the maximum incised surface was observed. We calculated the relative infarcted area as follows: infarcted area (%) = [hyperintensity in the lesion hemisphere (mm^2^) − hyperintensity in the nonlesion hemisphere (mm^2^)]*/*whole brain area (mm^2^) [22]. Survival rates were compared after 14 days (Figure 1(a)).

### 2.4. 2,3,5-Triphenyltetrazolium Hydrochloride (TTC) Staining

Rats underwent surgery as described above and were divided into two groups (Goreisan: *n* = 1, control: *n* = 1) and MRI was performed 24 h after surgery. Forty-eight hours after the surgery, rats were deeply anesthetized using ethyl ether (2.0%) and were transcardially perfused using phosphate-buffered saline (PBS) at 48 h after the surgery. Brain sections (2 mm thick) were prepared and stained with 2% TTC (Wako, Tokyo, Japan). Regions with active mitochondrial activity stain red, and less intensely stained regions are ischemic.

### 2.5. RNA Isolation from the Ischemic Rat Brain and Quantitative Reverse-Transcription PCR Analysis

Rats (P21) were divided into Goreisan (*n* = 6), nontreated (*n* = 6), and sham control (*n* = 6) groups (Figure 1(b)). After confirming the ischemic lesions using MRI, the rats were deeply anesthetized with ethyl ether, the brains were dissected 36 h after surgery (subacute phase of HIE), and 2 mm thick coronal sections were prepared. Total RNA was extracted from cerebral cortices in the ischemic lesion using ISOGEN (Nippon-gene, Tokyo, Japan) according to the manufacturer's instructions. Quantitative reverse-transcription PCR (qPCR) was performed as described previously [23]. The levels of mRNA expression were normalized to those of the glyceraldehyde 3-phosphate dehydrogenase* (Gapdh)* mRNA. The primer sequences were as follows: for AQP4: sense, 5′-gaatccagctcgatcctttg-3′, and antisense, 5′-cttcctttaggcgacgtttg-3′; for GAPDH: sense, 5′-gagacagccgcatcttcttg-3′, and antisense, 5′-tgactgtgccgttgaacttg-3′.

### 2.6. Immunohistochemistry

Rats were transcardially perfused using 4% (w/v) paraformaldehyde in PBS containing 2 mM MgCl_2_ and fixed brains were immersed overnight in PBS containing 15% sucrose. Brains were embedded in optimal cutting temperature compound NEG50 (Richard-Allan Scientific, MI, USA), frozen using dry ice/acetone, and cut into 10 *μ*m thick sections. Sections were reacted with the anti-AQP4 antibody diluted 1 : 200 in TBS-Tween-20 containing 0.1% bovine serum albumin (BSA) followed by incubation with a DyLight549-conjugated anti-mouse secondary antibody (Jackson ImmunoResearch Laboratories, West Grove, PA) in the same solution used for primary antibody incubation. The sections were stained with Hoechst 33342 (Sigma-Aldrich, St. Louis, MO) to visualize nuclear DNA.

### 2.7. Immunoblotting

**Figure 2:**
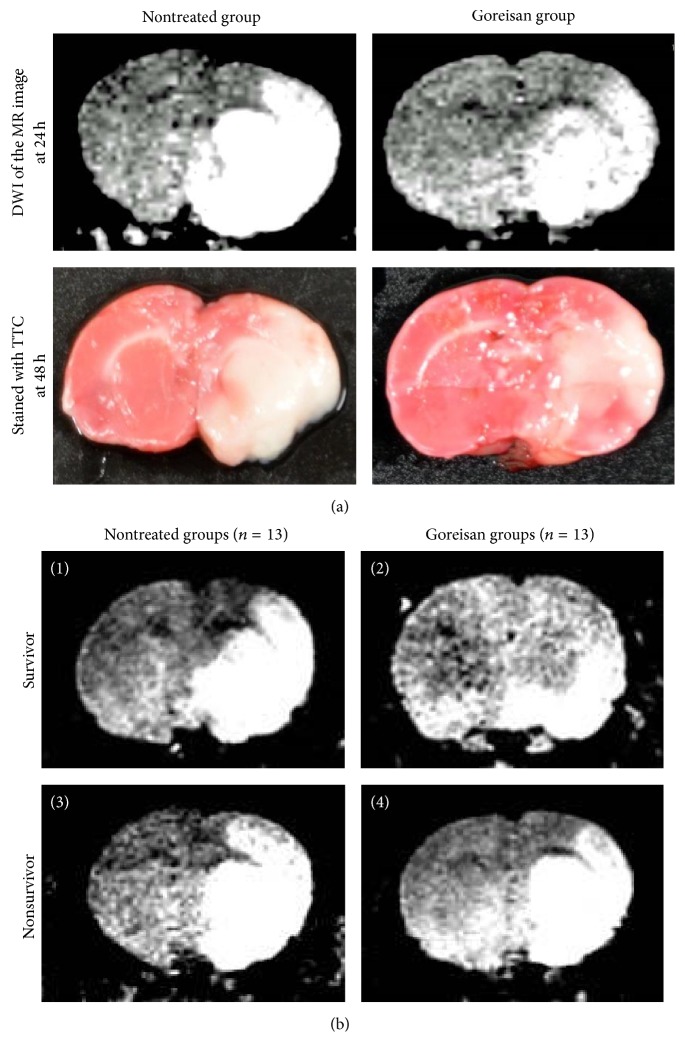
*Ischemic regions of right hemispheres identified using TTC staining and MRI*. (a) MR images were obtained using DW imaging (DWI) at 24 h. No TTC staining was observed in the ischemic region at 48 h. The MR imaging area and unstained TTC area overlapped in the lesioned rats. (b) The DWI of survivors in the nontreated group (1) or Goreisan group (2) and nonsurvivors in the nontreated group (3) or Goreisan group (4) at 24 h. MRI revealed that the lesion area was smaller in survivors from the Goreisan group compared with the other groups.

**Figure 3:**
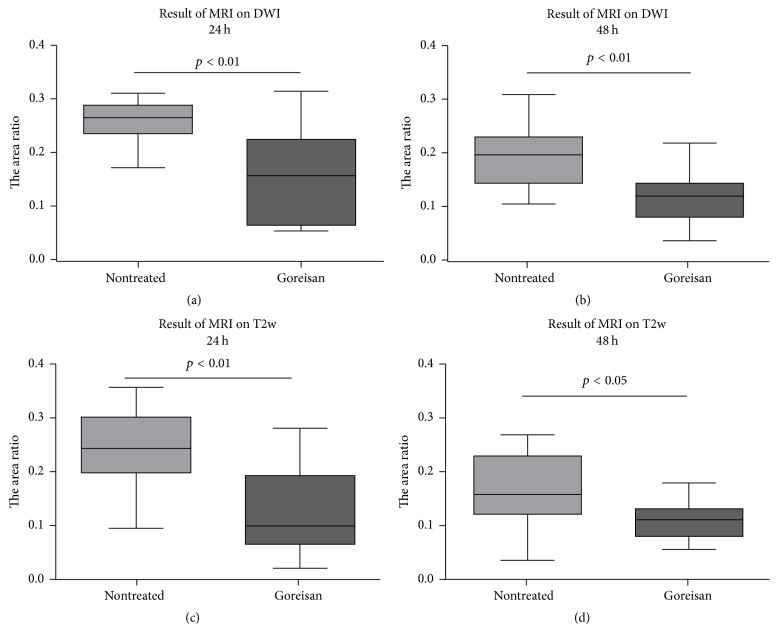
*Lesion area measured using MRI*. Ratios of the ischemic area to the total slice area in MRI for (a, b) DWI and (c, d) T2w are expressed as the median, range, and 25–75 percentiles. The MRI findings indicated that the lesion areas were significantly smaller in the Goreisan group than in the nontreated group at 24 and 48 h.

Immunoblotting was performed 12, 36, and 72 h after surgery. Rats (P21) were divided into Goreisan (*n* = 5) and nontreated (*n* = 5) groups at 12 h for the acute phase (Figure 1(c)-(1)), Goreisan (*n* = 5) and nontreated (*n* = 4) groups at 36 h for the subacute phase (Figure 1(c)-(2)), and Goreisan (*n* = 5) and nontreated (*n* = 5) groups at 72 h for the chronic phase (Figure 1(c)-(3)). Rat tissues were homogenized using Laemmli buffer and subjected to immunoblotting analysis as described elsewhere [24]. Antibodies specific for AQP4 and *β*-actin (ACTB) were from Santa Cruz Biotechnology, Inc. (Dallas, TX), and WAKO (Tokyo, Japan), respectively. Can Get Signal solution (Toyobo, Osaka, Japan) was used to enhance the signals, and the blots were visualized using alkaline phosphatase-labeled secondary antibodies (Promega, WI, USA) [25]. Immunoreactive bands were analyzed using ImageJ 1.43u software (Wayne Rasband, National Institutes of Health, Bethesda, MD). The densitometry data were normalized to the band intensities of ACTB.

### 2.8. Data Analysis

Data are presented as the mean ± standard error of the mean (SE), unless otherwise noted. GraphPad Prism Ver. 6.0 for Windows (GraphPad Software, San Diego, CA) was used for the analyses. Data were compared using one-way analysis of variance followed by Tukey's multiple comparisons test, the Mann–Whitney *U* test, and the unpaired *t-*test. The Kaplan–Meier method was used to compare survival rates, and survival outcomes were compared between groups using the log-rank test.

## 3. Results

### 3.1. Establishment of HIE in Rats

To study the effects of Goreisan on HIE in rats during the developmental stage equivalent to that of a child, we established a juvenile rat model of HIE by combined MCAO and CCAO. The induction of ischemic lesions in the right hemisphere was indicated by the loss of TTC staining and a strong MRI signal, which overlapped in the lesioned rats (Figure 2(a)).

### 3.2. Effects of Goreisan on Pathological Changes and Improved Survival Rates after HIE

**Figure 4:**
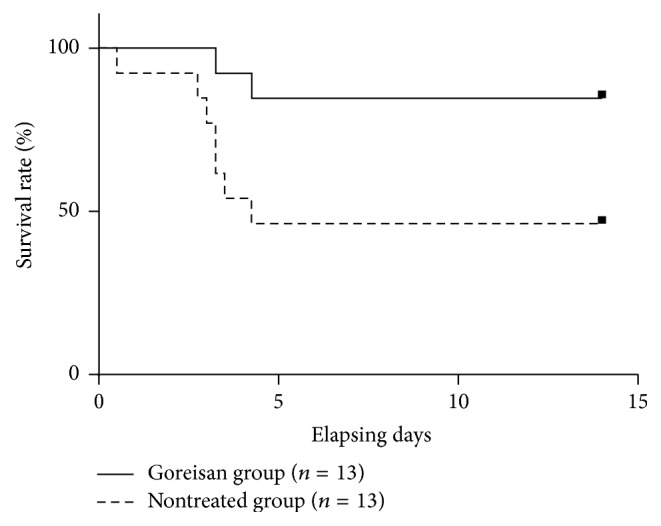
*Survival curves*. The survival rate of the Goreisan group was significantly higher than that of the nontreated group (*p* < 0.05).

To study the effects of Goreisan on the time-dependent changes in the HIE lesions, MRI evaluation was performed 24 h (Figure 2(b)) and 48 h after surgery. After 24 h, DWI revealed that the lesion area in the Goreisan group (median: 0.158, range: 0.056–0.313) was significantly smaller than in the nontreated group (0.264, 0.172–0.310; *p* < 0.01; Figure 3(a)). After 48 h, the lesion area of the Goreisan group (0.126, 0.043–0.225) was also smaller than that of the nontreated group (0.203, 0.111–0.315; *p* < 0.01; Figure 3(b)). T2w imaging revealed that the lesion area of the Goreisan group (0.102, 0.025–0.281) was significantly smaller than that of the nontreated group (0.244, 0.098–0.355) after 24 h (*p* < 0.01; Figure 3(c)) and after 48 h (Goreisan group: 0.113, 0.058–0.182; nontreated group: 0.160, 0.037–0.271; *p* < 0.05; Figure 3(d)). The survival rate was better in the Goreisan group than in the nontreated group (*p* < 0.05), indicating a beneficial effect of Goreisan on HIE in juvenile rats (Figure 4).

### 3.3. Goreisan Reduces AQP4 mRNA Levels in Ischemic Lesions during the Subacute Phase of HIE

**Figure 5:**
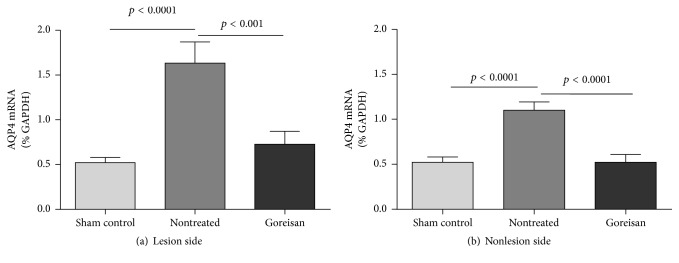
*AQP4 mRNA expression levels*. Aquaporin 4 (AQP4) mRNA levels 36 h after surgery revealed the inhibitory effects of Goreisan. In the lesion side (a), the level of AQP4 mRNA of the nontreated group was significantly higher than that in the sham control, and the level of AQP4 mRNA in the Goreisan group was significantly lower than that of the nontreated group. In the nonlesion side (b), the level of AQP4 mRNA of the nontreated group was significantly higher than that of the sham control, and the level of AQP4 mRNA in the Goreisan group was significantly lower than that of the nontreated group.

We used qPCR to measure the AQP4 expression 36 h after surgery. In the lesion side, the mean AQP4 mRNA level in the nontreated group (1.631 ± 0.238) was significantly higher than that in the sham control group (0.520 ± 0.058, *p* < 0.0001), and the mean AQP4 mRNA level in the Goreisan group (0.726 ± 0.143) was significantly lower than that in the nontreated group (*p* < 0.001; Figure 5(a)). In the nonlesion side, AQP4 mRNA level in the nontreated group (1.098 ± 0.094) was significantly higher than that in the sham control group (0.520 ± 0.058, *p* < 0.0001) and was significantly lower in the Goreisan group (0.519 ± 0.086) than in the nontreated group (*p* < 0.0001; Figure 5(b)). These results suggest that Goreisan inhibited AQP4 transcription during the subacute phase of HIE.

### 3.4. Goreisan Reduces AQP4 Protein Levels in the Ischemic Core in the Chronic Phase

**Figure 6:**
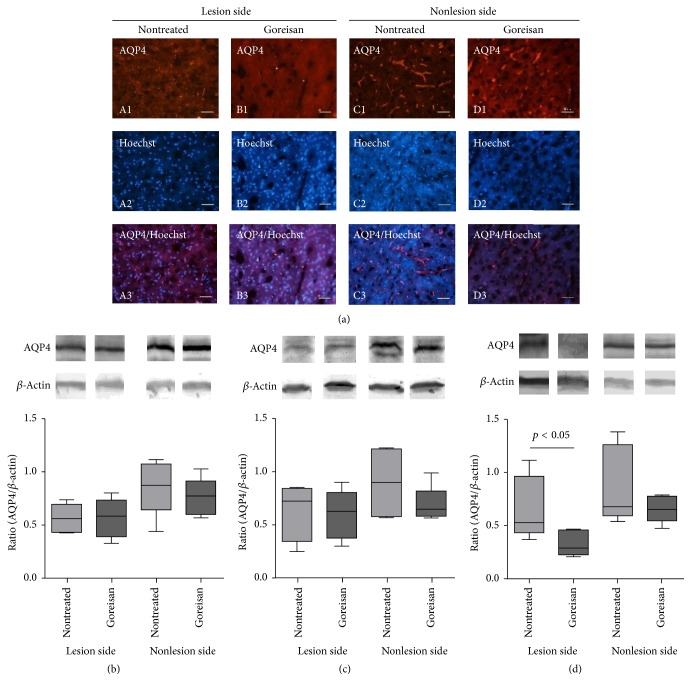
*AQP4 protein levels*. (a) Protein levels of AQP4 in a juvenile model of HIE induced brain injury. Immunostaining of AQP4 (1) in brain sections is shown together with counterstaining of nuclei by Hoechst 33342 (2). Merged images are shown in (3). The lesion side (A, B) versus nonlesion side (C, D) of nontreated (A, C) and Goreisan (B, D) groups is presented. Typical localization of AQP4 on astrocyte end-feet was observed in the nonlesion side (C1 and D1) [to a lesser degree in the Goreisan (D1) group] and at prominently reduced levels in the lesion side (A1 and B1). (b–d) Representative western blots of AQP4 and *β*-actin and their quantification at (b) 12 h, (c) 48 h, and (d) 72 h in the lesion and nonlesion sides. Data are expressed as the median, range, and 25–75 percentiles. No significant difference was observed between the Goreisan and nontreated groups at 12 h (b) or 48 h (c). However, Goreisan significantly decreased AQP4 protein levels in the lesion side at 72 h. AQP4 protein levels were comparable between the lesion and nonlesion side in the nontreated groups at 72 h (d).

AQP4 signal was prominently reduced in the ischemic core during the subacute phase (36 h after surgery) compared with that in the nonlesion side (Figure 6(a)) as previously reported [14]. The reduced AQP4 protein levels were comparable between the Goreisan treatment and control groups (Figure 6(a), lesion side, Goreisan), suggesting less prominent effects of Goreisan on AQP4 protein levels during the subacute phase. AQP4 staining was observed on end-foot-like structures of astrocytes (Figure 6(a), nonlesion side). Time course analyses of AQP4 protein levels by immunoblotting of brain homogenates revealed the upregulation of AQP4 in the nontreated lesion side at 72 h (0.524, 0.363–1.120) and suppression of upregulation in the Goreisan-treated lesion side (0.281, 0.198–0.462, *p* < 0.05 versus nontreated lesion side) (Figure 6(d)). Different AQP4 levels were not observed between nontreated and Goreisan-treated animals at 12 h (Figure 6(b)) and 36 h (Figure 6(c)), indicating that the different AQP4 protein levels observed between nontreated and Goreisan-treated groups followed the reduction in mRNA levels in the Goreisan-treated lesion side at 36 h (Figure 5).

## 4. Discussion

We developed a rat model of juvenile HIE, which we employed to demonstrate that Goreisan significantly suppressed cerebral edema and prolonged survival. During the subacute phase of HIE, Goreisan significantly suppressed the elevation of AQP4 mRNA levels in the lesion and nonlesion sides. Furthermore, Goreisan significantly suppressed AQP4 protein level in the chronic phase but did not have a significant effect in the acute or subacute phases. It is important to note that MCAO, which is effective for inducing HIE in adult rats, does not cause any pathological changes in juvenile rats. To overcome this problem, we induced surgical ischemic injury using MCAO and CCAO in juvenile rats, which exhibited ischemic lesions similar to those observed in the adult model by TTC staining and MRI, indicating that our rat HIE model is appropriate for analyzing the effect of Goreisan on juvenile HIE.

In this study, Goreisan improved the survival rates of the juvenile rats with HIE, possibly through preventing the development of ischemic lesions by suppressing brain edema. Very few studies have reported the effectiveness of Goreisan for treating cerebral edema associated with brain insults. Japanese clinicians have reported some case series of Goreisan use for chronic subdural hematoma and brain edema, mostly in Japanese journals [26, 27]. However, these data were limited because of the small number of patients and lack of any comparison with controls. A retrospective study in the Japan National Inpatient Database clarified a significant reduction in the requirement of subsequent surgery after burr-hole surgery to treat chronic subdural hematomas in patients who used Goreisan compared with patients who did not use Goreisan [12]. However, there have been no randomized placebo-controlled study showing the effect of Goreisan for cerebral edema associated with brain insults. In addition, the mechanism of the antiedematous effects of Goreisan is still unknown. Therefore, it is crucial to clarify the pathogenesis of cerebral edema and the effect of Goreisan on HIE with a rat model.

Brain edema is classified into the main pathological processes of cytotoxic edema, in which the prominent feature is the swelling of the cellular elements of the brain parenchyma, and vasogenic edema, in which increased vascular permeability leads to the accumulation of edema fluid in the extracellular spaces [28]. Cytotoxic and vasogenic mechanisms contribute to the pathogenesis of ischemic brain edema. AQP4 localizes to astrocytic end-feet that adhere to the astrocytic basal membrane. Together, these form the perivascular/Virchow-Robin space along with pericytes embedded in the vascular basement membrane that surrounds vascular endothelial cells, which form the BBB in postcapillary venules of the brain. In contrast, the BBB in brain capillaries are formed from the fusion of the astrocytic basal membrane and vascular basement membrane, which are wrapped around endothelial cells and pericytes accompanied by adherent astrocytic end-feet [29]. Cytotoxic edema is caused by reduced Na^+^-K^+^ ATPase activity, which increases Na^+^ concentrations inside astrocytes during energy failure induced by cerebral ischemia. This subsequently promotes the swelling of astrocytes by the influx of water caused by increased osmotic pressure via upregulated AQP4. Thus, cytotoxic edema is assumed to damage the BBB culminating in cytotoxic and vasogenic mixed edema. In contrast, intracerebral hemorrhage is accompanied by breakdown of the BBB, and vasogenic edema without astrocytic end-feet swelling precedes cytotoxic edema in astrocytes with upregulated AQP4, which is subsequently induced by increased movement of water into the interstitial fluid, suggesting mixed edema [30].

In the present study, Goreisan significantly suppressed AQP4 mRNA levels during the subacute phase of HIE and AQP4 protein levels during the chronic phase; however, AQP4 protein levels were not significantly altered during the acute and subacute phases. AQP4 mRNA levels in the lesion were higher than those in the sham control during the subacute phase, which is consistent with the findings of a previous report [16]. AQP4 protein levels have been reported to decrease during the subacute phase around 24 h and increase after 72 h (chronic phase) [14]. These findings suggest that Goreisan suppresses elevation of AQP4 mRNA levels in the lesion side during the subacute phase and may suppress AQP4 protein production, which in turn improves vasogenic edema during the chronic phase, culminating in improved survival rates.

The chronic phase is followed by the resolution phase when there is a net movement of water from brain to blood. In this phase, a reduced AQP4 level is a disadvantage rather than an advantage in improvement of edema. The precise time of onset of the resolution phase is debatable, but prominent resolution can be seen around day 7 after the onset of edema [31, 32]. Whether 72 h after MCAO surgery is in the resolution phase or not should be precisely defined, for example, by measuring AQP4 protein levels at later time points such as day 3, 7, and 14 in combination with MRI images. However, the improved survival rate, accompanied with suppressed AQP4 protein levels in edema lesions at 72 h, suggests that this time point might still be in the edema formation phase, at least in our animal model.

Goreisan has been suggested to prevent the water trafficking function of AQP4; therefore, decreased levels of AQP4 mRNA and protein following Goreisan treatment are a novel observation. However, Goreisan treatment did not change AQP4 levels during the acute phase, indicating that Goreisan does not have a direct effect on AQP4 gene transcription itself. Indeed, administration of Mn^2+^ and Mg^2+^, which are important components of Goreisan, to our animal model of edema produced no suppressive effects on AQP4 mRNA (data not shown). These results collectively suggest that the decreased AQP4 mRNA and protein levels are secondary events among various other changes to mRNAs and proteins that are induced by Goreisan treatment. Although being a secondary effect, AQP4 levels were influenced by Goreisan and correlated with an improved survival rate. We, therefore, propose one of the effects of Goreisan is inhibition of AQP4.

A limitation of our study is the oral administration of Goreisan 1 h before surgery, which was performed because immediate administration after surgery caused death due to respiratory suppression induced by abdominal distention. Increased levels of AQP4 are detected within 1 h after a brain insult [32]. Therefore, Goreisan was able to act through the entire process of the disease, assuming the medicine remained stable and active. In future studies, it will be important to determine the effects of Goreisan administration after injury, which would more accurately represent the clinical scenario.

In summary, our findings indicate that Goreisan is an effective antiedematous medication for the treatment of HIE in juvenile rats via the regulation of AQP4 expression and activity. Our findings support the consideration of Goreisan in the development of new prophylactic treatments for HIE-related cerebral edema, not only for juvenile HIE but also for patients of all ages.

## Figures and Tables

**Table 1:** Herbal medicines composing Goreisan.

Herbal medicine or plant name		(g)
Alisma Rhizome	*Alisma orientale* Juzepczuk	4.0
*Atractylodes lancea* Rhizome	*Atractylodes lancea De Candolle*	4.0
Polyporus Sclerotium	*Polyporus umbellatus* Fries	3.0
Poria Sclerotium	*Poria cocos* Wolf	3.0
Cinnamon bark	*Cinnamomum cassia* Blume	1.5
